# Intratumoural and Peritumoural Radiomics-Based Machine Learning Models for the Postoperative Survival Prediction in Oesophageal Squamous Cell Carcinoma

**DOI:** 10.1093/icvts/ivaf293

**Published:** 2026-03-10

**Authors:** Zhi-Qiang Deng, Chen Yang, Zhong-Feng Yan, Yue Li, Hong-Tao Tang, Hou-Dong Zuo, Jun-Jie Zhang, Wen-Long Hu, Yu-Yang Mao, Dai-Yuan Ma, Kai-Yuan Jiang, Hao-Ji Yan, Dong Tian

**Affiliations:** Department of Thoracic Surgery, West China Hospital, Sichuan University, Chengdu, 610041, China; College of Medical Imaging, North Sichuan Medical College, Nanchong, 637000, China; College of Clinical Medicine, North Sichuan Medical College, Nanchong, 637000, China; College of Clinical Medicine, North Sichuan Medical College, Nanchong, 637000, China; Department of Cardiovascular Surgery, West China Hospital, Sichuan University, Chengdu, 610041, China; Medical Imaging Key Laboratory of Sichuan Province, Department of Radiology, Affiliated Hospital of North Sichuan Medical College, Nanchong, 637000, China; College of Medical Imaging, North Sichuan Medical College, Nanchong, 637000, China; College of Clinical Medicine, North Sichuan Medical College, Nanchong, 637000, China; College of Clinical Medicine, North Sichuan Medical College, Nanchong, 637000, China; Department of Oncology, Affiliated Hospital of North Sichuan Medical College, Nanchong, Sichuan, 637000, China; Department of Surgery, Tohoku University Graduate School of Medicine, Sendai 980-8575, Japan; Department of General Thoracic Surgery, Juntendo University School of Medicine, Tokyo 113-8421, Japan; Department of Thoracic Surgery, West China Hospital, Sichuan University, Chengdu, 610041, China

**Keywords:** oesophageal squamous cell carcinoma, survival, radiomics, machine learning

## Abstract

**Objectives:**

This study aimed to develop and validate machine learning (ML) models to predict survival following oesophagectomy in oesophageal squamous cell carcinoma (ESCC) patients using intratumoural and peritumoural radiomic features.

**Methods:**

A retrospective analysis was conducted on ESCC patients with preoperative contrast-enhanced computed tomography who underwent oesophagectomy from June 2016 to January 2020. Patients were randomly assigned to training and test sets (8:2 ratio). Radiomic features were independently extracted from intratumoural and peritumoural regions. Cox regression, random survival forests (RSF), and gradient boosting decision tree (GBDT) were used for modelling. The performance of models was evaluated by discrimination and calibration.

**Results:**

The study included 443 patients, 354 in the training set and 89 in the test set. Peritumoural radiomic features predominated in the final selection, with 14 of 17 selected features originating from peritumoural region. The optimal GBDT model (the integrated area under the curve [iAUC]: 0.854; the integrated Brier score [iBS]: 0.160) using dual-region radiomic and clinical features outperformed other models, with a 1-year time-dependent area under the curve (tAUC) of 0.712 (95% CI, 0.655-0.738) and 3-year tAUC of 0.733 (95% CI, 0.655-0.805). It effectively stratified patients into high- and low-risk groups (*P *< .001).

**Conclusions:**

ML models using intratumoural and peritumoural radiomic features showed potential for predicting postoperative survival in ESCC patients, with the optimal GBDT model demonstrating effective risk stratification.

## GRAPHICAL ABSTRACT

**Figure:**
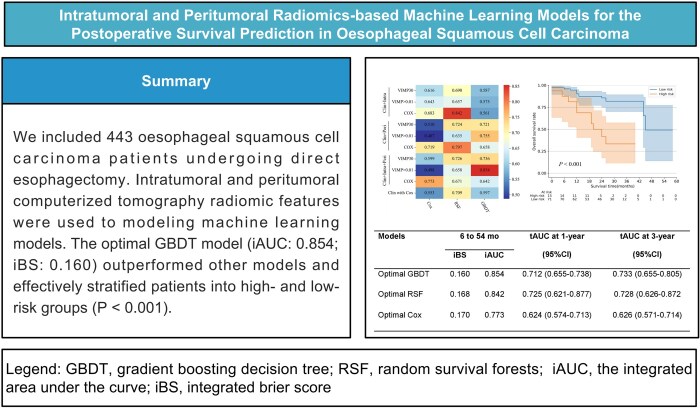

## INTRODUCTION

Oesophageal squamous cell carcinoma (ESCC) accounts for 90% of all oesophageal cancer cases in East Asia.^1^^,^^2^ Preoperative neoadjuvant therapy of ESCC has gained much attention but may cause adverse side effects.^3^^,^^4^ Thus, it is imperative to predict the postoperative survival of ESCC to confirm whether oesophagectomy alone is sufficient. For patients who are predicted to have favourable prognosis with direct surgery, choosing this approach could alleviate the side effects caused by neoadjuvant therapy. The eighth edition of the TNM staging system is commonly used for postoperative prognosis assessment in ESCC. Nevertheless, restricted pathological parameters and the heterogeneity of advanced tumours limited its effectiveness in clinical decision-making.^5^ Consequently, developing a novel prognostic tool based on preoperative data is warranted to assist clinicians in formulating preoperative therapeutic strategies.

Radiomics can extract high-throughput information from medical images, offering a detailed description of heterogeneous biological characteristics within a lesion.^6^^,^^7^ In ESCC, computed tomography (CT) radiomics has played a pivotal role in prognostic prediction.^8^^,^^9^ However, previous studies have primarily focused on intratumoural information to predict survival in ESCC, paying limited attention to the peritumoural region.^8^^,^^10^^,^^11^ Some studies found that peritumoural tissue biological information in ESCC correlates with local invasion and tumour growth. Thus, peritumoural radiomic features of ESCC may contain complementary and valuable prognostic information.^12^^,^^13^ The high-dimensional radiomic features require powerful analytical tools. Machine learning (ML) could mitigate the limitations of conventional survival analysis based on Cox regression.^10^^,^^14^^,^^15^ However, whether ML models based on intratumoural and peritumoural radiomics have similar potential for predicting postoperative survival remains unknown in ESCC. Therefore, this study aims to develop and validate ML models based on radiomic features from intratumoural and peritumoural regions to predict the postoperative survival of ESCC. Our research will focus on the following objectives: (1) assessing the incremental value of intratumoural and peritumoural radiomic features in fitting prognostic models for ESCC; (2) comparing the performance of ML models with a benchmark model fitted using Cox regression.

## PATIENTS AND METHODS

### Ethical statement

The study was approved by the ethics committees and review board of the Affiliated Hospital of North Sichuan Medical College (No. 2020ER181-1). Given its retrospective nature, the requirement for informed consent was waived. Any collection and storage of data from research participants for multiple and indefinite use were consistent with requirements outlined in the WMA Declaration of Taipei. The ethics committee approved the establishment and monitor ongoing use of databases. All case records were de-identified before analysis.

### Patients

Patients with ESCC who underwent contrast-enhanced CT scans before surgery at the Affiliated Hospital of North Sichuan Medical College between June 2016 and February 2020 were retrospectively enrolled (**Supplemental Methods**). The eighth edition of the American Joint Committee on Cancer (AJCC) and Union for International Cancer Control (UICC) oesophageal cancer staging system was used for pathological staging. The enrolled patients were randomly subclassified into a training set and a test set at a ratio of 8:2. Baseline characteristics of patients were collected (**Table 1**).

**Table 1.** Clinical Characteristics of Patients with ESCC

Characteristics	Patients, No. (%)		
	**All patients** **(*N* = 443)**	**Training set** **(*n* = 354)**	**Test set** **(*n* = 89)**
Age (years), median (range)	66 (45-83)	66 (45-83)	67 (49-80)
Sex			
Male	302 (68.2)	239 (67.5)	63 (70.8)
Female	141 (31.8)	115 (32.5)	26 (29.2)
BMI (kg/m^2^)	22.85 (3.84)	22.81 (3.93)	23.03 (3.46)
Preoperative complications			
No	258 (58.2)	206 (58.2)	52 (58.4)
Yes	185 (41.8)	148 (41.8)	37 (41.6)
Tumour location			
Upper	53 (12.0)	42 (11.9)	11 (12.4)
Middle	240 (54.2)	191 (54.0)	49 (55.1)
Lower	150 (33.9)	121 (34.2)	29 (32.6)
Preoperative T stage			
T1	160 (36.1)	124 (35.0)	36 (40.4)
T2	163 (36.8)	132 (37.3)	31 (34.8)
T3	101 (22.8)	83 (23.4)	18 (20.2)
T4a	19 (4.3)	15 (4.2)	4 (4.5)
Preoperative N stage			
N0	254 (57.3)	202 (57.1)	52 (58.4)
N1	77 (17.4)	62 (17.5)	15 (16.9)
N2	105 (23.7)	83 (23.4)	22 (24.7)
N3	7 (1.6)	7 (2.0)	0 (0.0)
TNM stage			
I	134 (30.2)	106 (29.9)	28 (31.5)
II	158 (35.7)	124 (35.0)	34 (38.2)
III	125 (28.2)	102 (28.8)	23 (25.8)
IV A	26 (5.9)	22 (6.2)	4 (4.5)
Preoperative haemoglobin	125.87 (14.08)	126.22 (13.86)	124.47 (14.96)
Preoperative AST	25.45 (27.66)	23.87 (11.74)	31.68 (56.92)

Continuous variables were presented as mean ± standard deviation and categorical variables as count and percentage. Preoperative complications typically involve chronic obstructive pulmonary disease, coronary heart disease and other diseases.

Abbreviations: AST: aspartate aminotransferase; BMI: body mass index; ESCC: oesophageal squamous cell carcinoma.

### Image delineation and feature extraction

**Figure 1.**
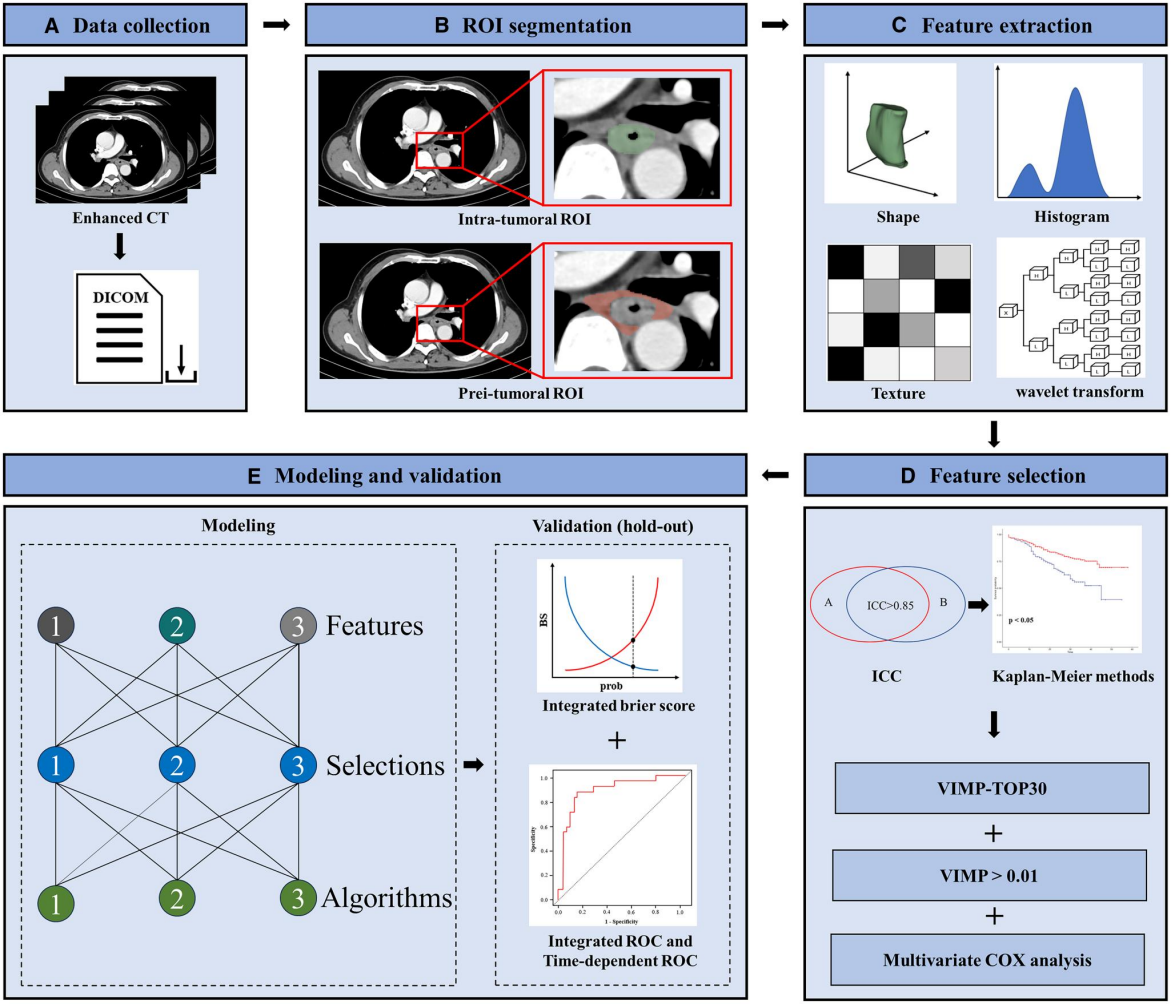
Flowchart of Radiomics Analysis. (A) Data collection; (B) ROI segmentation; (C) feature extraction; (D) feature selection; and (E) modelling and validation. Abbreviations: CT: computed tomography; ICC: intraclass correlation coefficient; intra: intratumoural features; Prei: peritumoural features; ROI: region of interest; VIMP: variable importance determined by bootstrap method repeated 1000 times; VIMP30: the top-30 features with VIMP sum; VIMP > 0.01: features with an average VIMP greater than 0.01.

Two radiologists (H.-T.T. and Z.-Q.D.) utilized 3 D Slicer (version 5.0.3) to independently manually delineate the regions of interest (ROIs) of intratumour and peritumour. The intratumoural ROI comprised the primary tumour tissue on the CT slices. The peritumoural ROI was defined as the lymph nodes and connective tissue surrounding the primary tumour on the CT slices, excluding the airway, aorta, spine, and superior vena cava.^11^ Subsequently, the PyRadiomics package version 1.2.0 in Python version 3.7 was used to extract radiomic features. A total of 851 radiomic features were extracted from intratumoural and peritumoural regions, respectively. The details of feature extraction and definition are shown in **Supplemental Methods**. All the extracted radiomic features were normalized before subsequent analysis (**Figure 1**).

### Feature selection

All statistical analyses were conducted using Python version 3.7 and R version 4.2.1. The selection of radiomic features was performed in 3 steps. Initially, features with a low interclass correlation coefficient (ICC) below 0.85 were excluded. Then, Kaplan-Meier analysis identified features correlated with patient survival; features demonstrating a *P*-value less than 0.05 were retained. Subsequent feature selection included 3 independent strategies. The first and second strategies utilized the gradient boosting decision tree (GBDT)-based variable importance (VIMP) measure, which was described in our previous study.^16^ To enhance the robustness of the process, bootstrapping resampling with 1000 repetitions was employed. In the first strategy, features exhibiting an average VIMP greater than 0.01 were included to fit the models. The second strategy selected the top 30 features based on the sum of VIMP values from 1000 repetitions. The third strategy employed multivariate Cox regression analysis to select significant features (*P *< .05). The feature selection procedures above were independently performed based on intratumoural features, peritumoural features, and dual-region features. Univariate and multivariate Cox regression were used for selecting clinical features, and factors with a *P *< .05 were selected. Finally, 9 groups of radiomic features (3 types of features × 3 selection methods) and 1 group of clinical features were selected for fitting models (**Figure 1**).

### Model development and validation

The “scikit-survival” package in Python was utilized to fit GBDT, random survival forest (RSF), and Cox regression models. Three types of selected intratumoural, peritumoural, and dual-region radiomic features were combined with clinical features to fit the models. A total of 27 radiomics-based models (3 × 3 × 3) were developed, including 3 combinations of radiomic and clinical features, 3 feature selection methods, and 3 modelling algorithms. Optimal hyperparameters were tuned using the grid search method (**Supplemental Methods**). Additionally, we developed clinical models using the same 3 modelling algorithms. Model validation was performed using the hold-out method.^17^ The performance of the models was assessed based on discrimination and calibration measures. Discrimination was assessed using the integrated area under the curve (iAUC) and the time-dependent area under the curve (tAUC). Calibration was evaluated using the integrated Brier score (iBS). The optimal cutoff value of the risk score outputted from the optimal ML model was confirmed using a log-rank analysis, identifying the value corresponding to the maximum survival difference between groups. Patients were classified into a high- or low-risk group according to the cutoff value. Additional methodological details are shown in the **Supplemental Methods**. The workflow of the radiomics analysis is shown in **Figure 1**. All statistical tests were 2-tailed, with the significance level set at *P *< .05.

## RESULTS

### Characteristics of patients

A total of 443 patients were included, with a median age of 66 years (range: 45-83), and 302 patients (68.2%) were male. The middle thoracic oesophagus was the most common tumour location (54.2%), followed by the lower thoracic oesophagus (33.9%). The highest proportions of preoperative clinical T stage, N stage, and TNM stage were T2 stage (163 patients [36.8%]), N0 stage (254 patients [57.3%]), and stage II (158 patients [35.7%]), respectively. The patients were randomly divided into a training set (354 patients) and a test set (89 patients) at an 8:2 ratio. The mean (SD) follow-up time in this cohort was 48 (20) months. Detailed characteristics of the patients in the 3 datasets are provided in **Table 1**.

### Feature selection

After excluding features with ICC below 0.85, 654 intratumoural features and 614 peritumoural features were selected. The Kaplan-Meier analysis selected 330 intratumoural features and 470 peritumoural features for subsequent analysis. For the dual-region radiomic feature selection, 17 features exhibiting a VIMP > 0.01 were selected (**Figure S1**), and Cox regression analysis confirmed 13 radiomic features with *P* < 0.05 (**Table S1**). Peritumoural features account for the majority in the selection of all methods (VIMP > 0.01: 14/17; VIMP-top30: 22/30; COX: 7/13) (**Table S1**). The Cox regression also selected 4 clinical features for modelling: preoperative complications, T stage, haemoglobin, and aspartate aminotransferase (AST) (**Table 2**).

**Table 2.** Univariate and Multivariate Cox Analysis for the Selection of Clinical Features

Characteristics	Univariate analysis			Multivariate analysis		
	HR (95% CI)		*P* value	HR (95% CI)		*P* value
Age (years)	1.011	(0.997-1.025)	.113			
Sex			.515			
Male			[Reference]			
Female	1.069	(0.875-1.307)	.515			
BMI (kg/m^2^)	1.014	(0.985-1.043)	.358			
Preoperative complications			.017*			.008*
No			[Reference]			[Reference]
Yes	1.267	(1.043-1.540)	.017*	1.301	(1.070-1.582)	.008*
Tumour location			.338			
Upper			[Reference]			
Middle	1.005	(0.746-1.354)	.973			
Lower	1.165	(0.851-1.597)	.341			
Preoperative T stage			<.001*			<.001*
T1			[Reference]			[Reference]
T2	1.443	(1.158-1.799)	.001*	1.445	(1.159-1.803)	.001*
T3	0.834	(0.649-1.073)	.159	0.870	(0.675-1.120)	.280
T4a	1.515	(0.940-2.441)	.088	1.710	(1.057-2.768)	.029
Preoperative N stage			.065			
N0			[Reference]			
N1	1.421	(1.099-1.838)	.007*			
N2	1.115	(0.886-1.403)	.354			
N3	1.131	(0.533-2.399)	.749			
TNM stage			.086			
I			[Reference]			
II	0.869	(0.119-6.325)	.890			
III	1.299	(0.182-9.279)	.794			
IVA	1.212	(0.169-8.669)	.848			
Preoperative haemoglobin	0.991	(0.985-0.998)	.013*	0.992	(0.985-0.999)	.026*
Preoperative AST	1.008	(1.005-1.012)	<.001*	1.008	(1.004-1.012)	<.001*

* *P *< 0.05. Preoperative complications mainly include chronic obstructive pulmonary disease, pulmonary tuberculosis, pneumoconiosis, pulmonary nodules, hypertension, coronary heart disease, anaemia, and other diseases.

Abbreviations: AST: aspartate aminotransferase; BMI: body mass index; HR: hazard ratio.

### Performance of the single-region models

**Figure 2.**
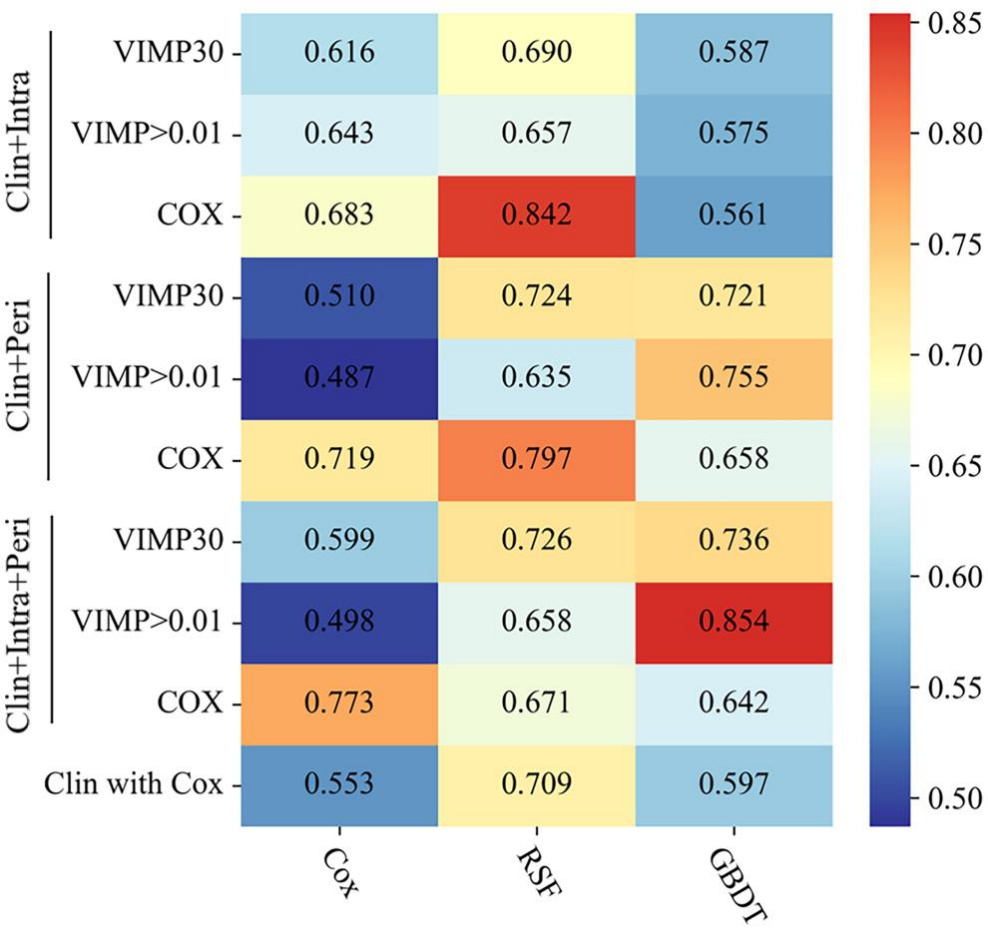
The Performance of 30 Different Models Developed Using Different Combinations of Features, Feature Selection Methods, and Modelling Algorithms. Those heatmaps showed the integrated area under the curve of each modelling algorithm (columns) with each feature selection method (rows). Abbreviations: Clin: clinical features; Clin with Cox: clinical features selected by Cox regression; GBDT: gradient boosting decision tree; Intra: intratumoural features; Peri: peritumoural features; RSF: random survival forests; VIMP: variable importance; VIMP30: the top-30 features with VIMP sum; VIMP > 0.01: features with an average VIMP greater than 0.01.

Among the 9 models integrating intratumoural and clinical features, only 1 exhibited an iAUC value greater than 0.7 (range: 0.842-0.842). In contrast, 5 of the 9 models developed with peritumoural and clinical features achieved iAUCs greater than 0.7 (range: 0.719-0.797) (**Figure 2**). The RSF model developed with features selected by Cox regression presented the best performance in models of the 2 regions. The optimal clinical-intratumoural RSF model outperformed the optimal clinical-peritumoural RSF model, with iAUCs of 0.842 (iBS: 0.168) and 0.797 (iBS: 0.175), respectively. Similar results were found in the prediction of 1- and 3-year survival (**Tables S2-S4**).

### Performance of the intra- and peritumoural models

**Figure 3.**
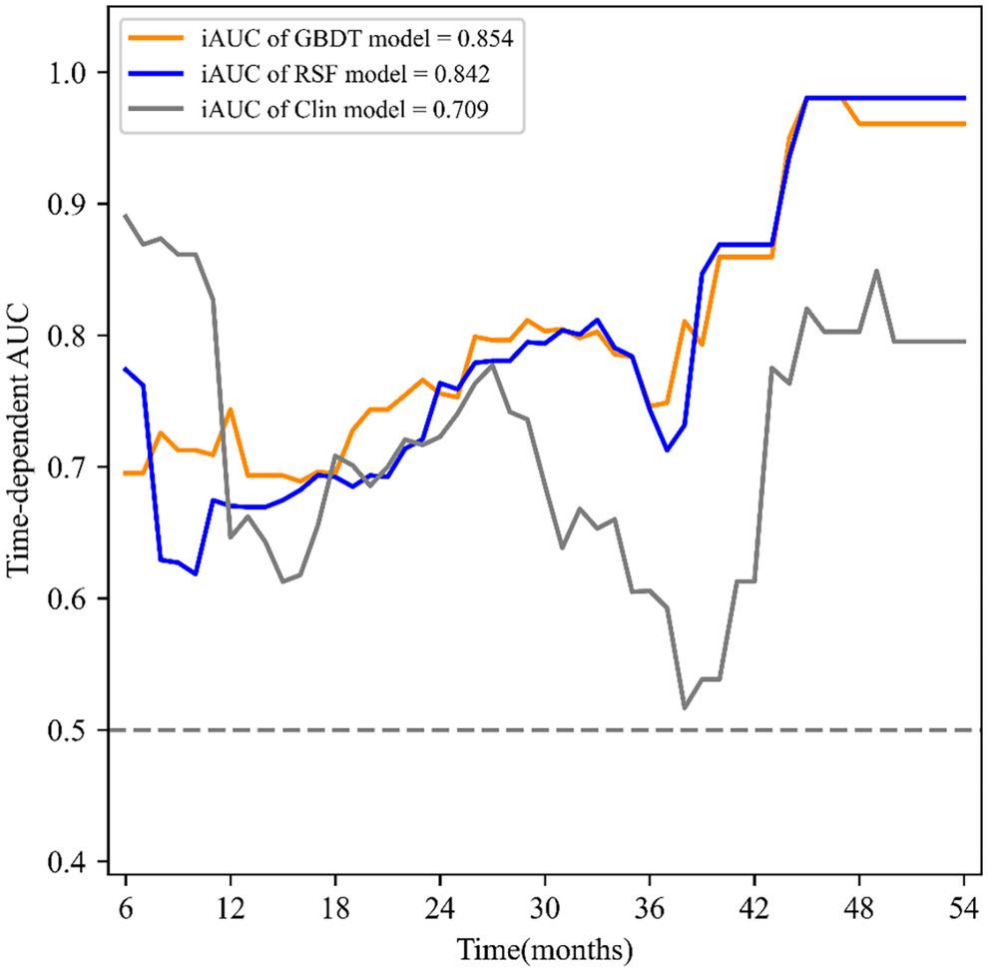
Time-Dependent ROC of the Optimal GBDT and RSF Models and Clinical RSF Model in the Test Cohort. Abbreviations: GBDT: gradient boosting decision tree; iAUC: the integrated area under the curve; RSF: random survival forest; VIMP: variable importance; VIMP > 0.01: features with an average VIMP greater than 0.01.

**Figure 4.**
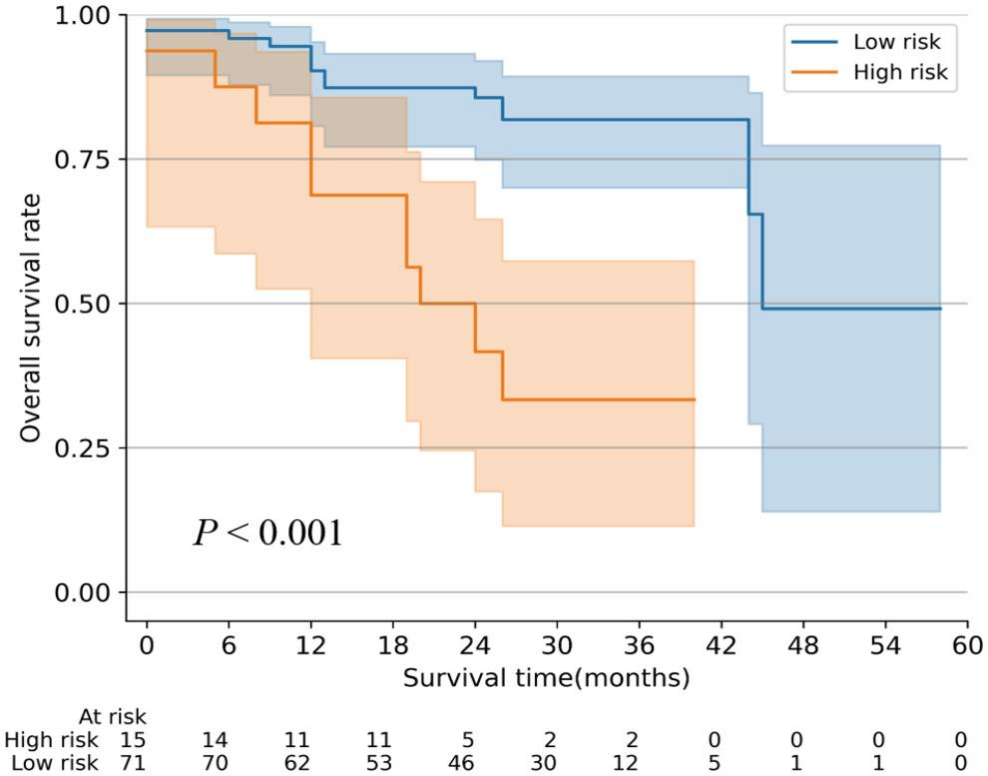
Survival Curves for ESCC Patients after Surgery with Risks Stratified in the Test Set. Abbreviations: ESCC: oesophageal squamous cell carcinoma; GBDT: gradient boosting decision tree.

Among the 9 models fitted with intra- and peritumoural radiomic and clinical features, 4 exhibited an iAUC exceeding 0.7. The GBDT model, integrating intra- and peritumoural radiomic features (VIMP > 0.01) with clinical features (preoperative complications, preoperative T stage, haemoglobin, and AST), demonstrated the best performance (**Figure 2**). The resulting optimal iAUC was 0.854, with an iBS of 0.160, better than the optimal single-region and Cox regression models (**Figure S2, Table 3**). In addition, it outperformed the optimal clinical-intratumoural RSF model and the clinical RSF model that was developed with clinical features alone (**Figure 4**). After the optimal GBDT model outputting the risk scores, the log-rank test determined a cutoff value of 0.45 for risk classification. The high-risk and low-risk groups show a significant difference in survival (*P *< .001) (**Figure 3, Table S5**). The optimal GBDT model also exhibited favourable performance in predicting survival at specific postoperative time points, with a 1-year tAUC of 0.712 (95% CI, 0.656-0.738) and a 3-year tAUC of 0.733 (95% CI, 0.656-0.805) (**Table 3**). The RSF model, based on only clinical features, exhibited the best performance for predicting 1-year postoperative survival (tAUC 0.765 [95% CI, 0.770-0.926]) (**Table S6**). Furthermore, we validated the optimal GBDT model on T1 and T2-4a stage subgroups (**Table S7**).

**Table 3.** Validation of the Optimal GBDT, RSF, and Cox Regression Models

Models	6 to 54 mo		**tAUC at 1-year** **(95% CI)**	**tAUC at 3-year** **(95% CI)**
	iBS	iAUC		
Optimal GBDT	0.160	0.854	0.712 (0.655-0.738)	0.733 (0.655-0.805)
Optimal RSF	0.168	0.842	0.725 (0.621-0.877)	0.728 (0.626-0.872)
Optimal Cox	0.170	0.773	0.624 (0.574-0.713)	0.626 (0.571-0.714)

The optimal GBDT model used clinical features and intra- and peritumoural features via VIMP > 0.01. The optimal RSF model used clinical features and peritumoural features via Cox regression. The optimal Cox model used clinical features and intra- and peritumoural features via Cox regression.

Abbreviations: CI: confidence interval; GBDT: gradient boosting decision tree; iAUC: the integrated area under the curve; iBS: integrated brier score; RSF: random survival forest; tAUC: time-dependent area under the curve.

## DISCUSSION

In this study, intratumoural and peritumoural CT radiomic features were combined with preoperative clinical features to develop and validate Cox regression and ML models for predicting survival in ESCC patients after surgery. The key findings are summarized as follows. First, peritumoural radiomic features demonstrated significant value in predicting the postoperative survival of ESCC, and incorporating them into the modelling process offers incremental value. Second, models employing ML algorithms tend to show better performance than those fitted using the traditional Cox regression.

The intratumoural tissue, reflecting tumour heterogeneity, contains a large amount of prognostic information. Nowadays, increasing attention has been paid to peritumoural tissue in ESCC, which could reflect changes in tumour growth, invasion, and immune response. Kam et al^12^ identified an infiltration of proliferating B cells in ESCC, which were absent within the tumour, confirming the association of hyperproliferative B cells with poor survival. Nonetheless, these changes are challenging to visualize through CT images. Radiomics facilitates the quantitative assessment of microenvironmental heterogeneity.^6^ In this study, peritumoural radiomic features of ESCC play a vital role in predicting postoperative survival. When selecting peritumoural and intratumoural radiomic features, 3 feature selection strategies consistently indicated that peritumoural radiomic features constituted the majority of the modelling features.

Intratumoural region retains significant prognostic value, numerous studies focusing on intratumoural CT radiomics in ESCC have demonstrated the importance of radiomics in prognostic prediction.^8^^,^^18^ However, studies of the peritumoural region remain scattered. Hu et al^11^ utilized peritumoural radiomics to predict post-pathological complete response to neoadjuvant chemoradiation in ESCC, finding peritumoural models were not significantly better than intratumoural ones. Consistent with prior findings, we did not demonstrate a significant superiority of peritumoural models over intratumoural models. This could be attributed to several biological and technical factors. First, intratumoural features directly reflect core biological characteristics of tumour cells, such as proliferation, necrosis, and heterogeneity. Second, the complex anatomy surrounding the oesophagus poses challenges for accurate delineation. Indeed, the delineation of the peritumoural ROI in this study depended on the expertise of radiologists. Third, the prognostic value of peritumoural regions is closely associated with tumour invasion depth. However, T1-stage ESCC often shows limited invasion into the peritumoural tissues, primarily within the oesophageal mucosa or submucosa.^19^ For advanced ESCC, tumour invasion extends to peritumoural tissues, the blurred tumour boundaries lead to overlapping intratumoural and peritumoural radiomic features.

ML can handle high-dimensional and complex data, surpassing Cox regression models in capturing nonlinear relationships within data.^20^ Our previous studies have demonstrated the effectiveness of the RSF algorithm, which outperforms the Cox regression model.^16^^,^^17^ A study developed an RSF model using clinical data for oesophageal cancer, achieving a tAUC of 0.839 in predicting 5-year survival.^21^ Consistent with their findings, the optimal RSF model in our study exhibited favourable performance, achieving an iAUC of 0.842. Moreover, we employed the GBDT algorithm for model construction, achieving optimal performance. The GBDT algorithm has advantages like converging to local optima and mitigating overfitting risk through a small learning rate and numerous iterations.^22^ In our study, the GBDT model surpassed other models and demonstrated satisfactory predictive ability for 1- and 3-year survival. Additionally, the optimal GBDT model effectively categorized patients into high and low-risk groups, which is vital in preoperative risk assessment and patient management strategies. Thus, for ESCC patients with a high risk of poor survival, preoperative neoadjuvant therapy was strongly recommended, even if they are initially staged as resectable.

This study presents several limitations. First, this is a single-centre retrospective study with a small sample size, which could potentially introduce selection biases and lead to wide confidence intervals. The models were only evaluated through internal validation in the test set, conducting large-scale and multicentre studies is necessary. Second, we enrolled both early- and advanced-stage patients, restricting the model’s applicability to stage-specific decisions. Third, none of the patients in this study received neoadjuvant therapy, which may limit the extrapolation of the results to ESCC patients who received neoadjuvant therapy. Fourth, the relatively short follow-up time may underestimate the long-term recurrence risk; future studies should verify the long-term performance of the model. Notably, the radiomics models were built based on single-phase CT. Integrating the temporal dynamic information from serial CT scans or multimodal features of PET-CT may optimize the predictive performance. In addition, an online real-time tool for ESCC survival prediction that fits clinical scenarios is expected to be developed based on our model.

## CONCLUSION

The present study employed ML-based radiomics of intratumoural and peritumoural regions to preoperatively predict the survival of ESCC patients. Peritumoural radiomic features were identified as crucial in predicting ESCC patients’ survival. The optimal GBDT model exhibited promising results in survival prediction and patient risk stratification. These findings showed the significance of intratumoural and peritumoural radiomics in prognosticating the survival of ESCC patients.

## Supplementary Material

ivaf293_Supplementary_Data

## Data Availability

The data will be shared on reasonable request to corresponding author.
