# Supplementary material for: Intratumoural and Peritumoural Radiomics-Based Machine Learning Models for the Postoperative Survival Prediction in Oesophageal Squamous Cell Carcinoma
Source: Interdiscip Cardiovasc Thorac Surg. 2026 Mar 10;41(3):ivaf293. doi: 10.1093/icvts/ivaf293 (PMC12987764; doi:10.1093/icvts/ivaf293)
Supplement: ivaf293_Supplementary_Data [file ivaf293_supplementary_data.docx]

**Contents**

**Supplemental Methods**

**Supplementary Table 1.** The intra- and peritumoral radiomic features selected by different feature selection strategies.

**Supplementary Table 2.** Validation of Cox, RSF and GBDT models based on intratumoral radiomic and clinical features.

**Supplementary Table 3.** Validation of Cox regression, RSF and GBDT models based on peritumoral radiomic and clinical features.

**Supplementary Table 4.** Validation of Cox models, RSF models and GBDT models based on intratumoral, peritumoral radiomic, and clinical features.

**Supplementary Table** **5.** Key clinical and radiomic modeling features of high-risk and low-risk groups.

**Supplementary Table 6.** Validation of the optimal RSF model based on clinical features alone.

**Supplementary Table 7.** Validation of the optimal GBDT model on T1 and T2-4a stage subgroups.

**Supplementary Figure 1.** The intra- and peritumoral radiomic features selected by the method of VIMP > 0.01.

**Supplementary Figure 2.** Time-dependent ROC of the optimal GBDT and Cox regression models in the test set.

**Supplemental Methods**

**1. Patient selection**

Patients with esophageal squamous cell carcinoma (ESCC) who underwent contrast -enhanced computed tomography (CT) before direct surgery were initially considered. Those with poor-quality CT images, a llack of much clinical data, or who had received postoperative adjuvant therapy were excluded. Ultimately, 443 patients were included in the study.

**2. Image delineation and feature extraction**

The CT images were resampled to a voxel size (3×3×3) using linear interpolation and the bin width was set to 25 during feature extraction. The extracted radiomic features included 14 first-order features, 18 shape features, 75 texture features, and 744 wavelet-transformed features in this study. First-order statistics describe the distribution of voxel intensities within the image region defined by the mask through commonly used and basic metrics. Shape features included descriptors of the two-dimensional and three-dimensional size and shape of the region of interests (ROI). These features are independent from the gray level intensity distribution in the ROI and are therefore only calculated on the non-derived image and mask. Texture features mainly describe the spatial relationship between pixels. Wavelet features are multiscale features obtained after wavelet transformation of the images, which reflects the change rate of the pixel value in the frequency domain. Missing values for all variables were handled using multiple imputation via the mice package in R 4.2.1, with 5 iterations to generate and pool complete datasets.

**3. Model development**

The hyperparameters for the optimal GBDT model were determined using the grid search method. Some key hyperparameters were as follows: loss = “coxph”, learning rate = 0.1, maximum tree depth = 4, maximum leaf nodes = 4, minimum samples leaf nodes = 2, minimum samples splits = 3，minimum weight fraction leaf = 0.2，and number of estimators = 90.

**4. Model validation**

Discrimination was assessed using the integrated area under the curve (iAUC), where higher values indicated greater discriminative ability. Calibration was evaluated using the integrated Brier score (iBS) that ranges from 0 to1, with values closer to 0 indicating better calibration. The iAUC and iBS were assessed every six months within the 6-54-month timeframe. The time-dependent area under the curve (tAUC) evaluated the performance of the model at specific time points (one and three years). In addition, the optimal risk score cutoff value divided patients into high- and low-risk groups. The survival and differences between two groups were evaluated using the Kaplan-Meier method and log-rank analysis. The risk stratification capability can be further demonstrated.

**Supplementary Table 1.** The intra- and peritumoral radiomic features selected by different feature selection strategies.

| **Feature selection strategy** | **Selected features** |
| --- | --- |
| VIMtop-30 | peri_wavelet_LLL_firstorder_Energy  peri_wavelet_LHH_gldm_Dependence Non-Uniformity Normalized intra_wavelet_LLH_firstorder_Median  peri_original_gldm_Dependence Variance  peri_wavelet_LLL_firstorder_Total Energy  peri_original_glszm_Large Area Low Gray Level Emphasis peri_wavelet_LHH_glcm_Inverse Variance  peri_wavelet_LLL_glrlm_Run Entropy  peri_wavelet_HLL_glcm_Imc1  peri_wavelet_LLH_glszm_Gray Level Non-Uniformity Normalized peri_wavelet_LLH_gldm_Small Dependence Low Gray Level Emphasis peri_wavelet_LLH_glszm_Gray Level Non-Uniformity  intra_wavelet_HLH_gldm_Small Dependence Low Gray Level Emphasis  peri_wavelet_HLH_glszm_Size Zone Non-Uniformity  peri_wavelet_HLH_glszm_Gray Level Non-Uniformity peri_wavelet_LLH_firstorder_10Percentile  peri_wavelet_HHH_glrlm_Low Gray Level Run Emphasis peri_wavelet_LLL_gldm_Dependence Entropy  intra_wavelet_HHL_glszm_Small Area Emphasis  intra_wavelet_HLL_glcm_Maximum Probability  intra_wavelet_LLH_gldm_Dependence Variance  peri_wavelet_LLH_firstorder_Energy  peri_wavelet_HHH_glcm_Correlation  peri_wavelet_LHH_firstorder_Median  peri_original_firstorder_10Percentile  intra_wavelet_HHH_glcm_Sum Average  intra_wavelet_HHH_glcm_Joint Average  peri_original_gldm_Dependence Non-Uniformity  intra_wavelet_HHL_firstorder_Median  intra_wavelet_HLL_glcm_Id |
| Cox | peri_original_shape_Minor AxisLength  peri_wavelet_LLH_glrlm_Short Run Emphasis  peri_wavelet_LHL_firstorder_Median  peri_wavelet_HLL_firstorder_Maximum  peri_wavelet_HHL_firstorder_Skewness  peri_wavelet_HHH_gldm_Dependence Variance  peri_wavelet_LLL_glrlm_Run Variance  intra_original_glcm_Idn  intra_wavelet_LHH_gldm_Large Dependence High Gray Level Emphasis  intra_wavelet_LHH_glrlm_Long Run High Gray Level Emphasis intra_wavelet_LHH_glszm_Small Area Low Gray Level Emphasis  intra_wavelet_HLL_glrlm_Short Run High Gray Level Emphasis  intra_wavelet_LLL_firstorder_90Percentile |

The intra- and peritumoral radiomic features were selected by VIMPtop-30 and Cox regression methods. LLL, Low-low-low; LHH, Low-high-high; LLH, Low-low-high; GLDM, Gray Level Dependence Matrix; GLSZM, Gray Level Size Zone Matrix; GLCM, Gray Level Co-occurrence Matrix; GLRLM, Gray Level Run Length Matrix; HLL, High-low-low; HLH, High-low-high; LHH, low-high-high; HLL, high-low-low; HLH, high-low-high.

**Supplementary Table 2.** Validation of Cox, RSF and GBDT models based on intratumoral radiomic and clinical features.

| **Clin+intra** | **6-54mo iBS** | | | **6-54mo iAUC** | | | **tAUC at 1year(95%CI)** | | | **tAUC at 3year(95%CI)** | | |
| --- | --- | --- | --- | --- | --- | --- | --- | --- | --- | --- | --- | --- |
|  | **Cox** | **RSF** | **GBDT** | **Cox** | **RSF** | **GBDT** | **Cox** | **RSF** | **GBDT** | **Cox** | **RSF** | **GBDT** |
| VIMP30 | 0.195 | 0.181 | 0.191 | 0.616 | 0.690 | 0.587 | 0.424  (0.399-  0.448) | 0.655  (0.562-  0.910) | 0.411  (0.374-  0.468) | 0.457  (0.415-  0.577) | 0.638  (0.553-  0.910) | 0.437  (0.374-  0.541) |
| VIMP>0.01 | 0.198 | 0.183 | 0.196 | 0.643 | 0.657 | 0.575 | 0.500  (0.470-  0.540) | 0.515  (0.441-  0.657) | 0.460  (0.390-  0.507) | 0.506  (0.471-  0.602) | 0.545  (0.451-  0.661) | 0.500  (0.403-  0.613) |
| COX | 0.180 | **0.168** | 0.187 | 0.683 | **0.842** | 0.561 | 0.446  (0.411-  0.499) | **0.725**  **(0.621-**  **0.877)** | 0.702  (0.621-  0.837) | 0.476  (0.422-  0.648) | **0.728**  **(0.626-**  **0.872)** | 0.680  (0.601-  0.837) |

VIMP: Variable importance; iBS, integrated brier score; iAUC, the integrated area under the curve; tAUC, time-dependent area under the curve; CI, confidence interval; RSF, random survival forest; GBDT, gradient boosting decision tree.

**Supplementary Table 3.** Validation of Cox regression, RSF and GBDT models based on peritumoral radiomic and clinical features.

| **Clin+peri** | **6-54mo iBS** | | | **6-54mo iAUC** | | | **tAUC at 1year(95%CI)** | | | **tAUC at 3year**  **(95%CI)** | | |
| --- | --- | --- | --- | --- | --- | --- | --- | --- | --- | --- | --- | --- |
|  | **Cox** | **RSF** | **GBDT** | **Cox** | **RSF** | **GBDT** | **Cox** | **RSF** | **GBDT** | **Cox** | **RSF** | **GBDT** |
| VIMP30 | **0.156** | 0.174 | 0.187 | 0.510 | 0.724 | 0.721 | 0.521  (0.466-  0.557) | 0.655  (0.556-  0.712) | 0.599  (0.434-  0.693) | 0.517  (0.455-  0.556) | 0.661  (0.567-  0.712) | 0.593  (0.434-  0.693) |
| VIMP>0.01 | 0.196 | 0.177 | 0.166 | 0.487 | 0.635 | 0.755 | 0.506  (0.447-  0.538) | 0.679  (0.619-  0.718) | **0.759**  **(0.713-**  **0.799)** | 0.511  (0.431-  0.578) | 0.693  (0.633-  0.738) | **0.742**  **(0.701-**  **0.799)** |
| COX | 0.179 | 0.175 | 0.186 | 0.719 | **0.797** | 0.657 | 0.573  (0.562-  0.621) | 0.717  (0.627-  0.844) | 0.726  (0.654-  0.825) | 0.594  (0.571-  0.698) | 0.716  (0.636-  0.844) | 0.724  (0.654-  0.825) |

VIMP: Variable importance; iBS, integrated brier score; iAUC, the integrated area under the curve; tAUC, time-dependent area under the curve; CI, confidence interval; RSF, random survival forest; GBDT, gradient boosting decision tree.

**Supplementary Table 4.** Validation of Cox models, RSF models and GBDT models based on intratumoral, peritumoral radiomic, and clinical features.

| **All** | **6-54mo iBS** | | | **6-54mo iAUC** | | | **tAUC at 1year(95%CI)** | | | **tAUC at 3year(95%CI)** | | |
| --- | --- | --- | --- | --- | --- | --- | --- | --- | --- | --- | --- | --- |
|  | **Cox** | **RSF** | **GBDT** | **Cox** | **RSF** | **GBDT** | **Cox** | **RSF** | **GBDT** | **Cox** | **RSF** | **GBDT** |
| VIMP30 | 0.173 | 0.146 | **0.137** | 0.599 | 0.726 | 0.736 | 0.458  (0.357-  0.489) | 0.470  (0.284-  0.577) | 0.543  (0.425-  0.674) | 0.553  (0.358-  0.635) | 0.624  (0.440-  0.723) | 0.677  (0.539-  0.773) |
| VIMP>0.01 | 0.205 | 0.185 | 0.160 | 0.498 | 0.658 | **0.854** | 0.563  (0.511-  0.607) | 0.675  (0.626-  0.704) | 0.712  (0.655-  0.738) | 0.538  (0.471-  0.607) | 0.683  (0.638-  0.713) | **0.733**  **(0.655-**  **0.805)** |
| COX | 0.170 | 0.178 | 0.184 | 0.773 | 0.671 | 0.642 | 0.624 | 0.732 | **0.744** | 0.626 | 0.702 | 0.700 |
|  |  |  |  |  |  |  | (0.574-  0.713) | (0.635-  0.922) | **(0.670-**  **0.880)** | (0.571-  0.714) | (0.640-  0.922) | (0.629-  0.869) |

VIMP: Variable importance; iBS, integrated brier score; iAUC, the integrated area under the curve; tAUC, time-dependent area under the curve; CI, confidence interval; RSF, random survival forest; GBDT, gradient boosting decision tree.

**Supplementary Table 5.** Key clinical and radiomic modeling features of high-risk and low-risk groups.

| Features | Patients, No. (%) | | |
| --- | --- | --- | --- |
|  | Low risk(n=73) | High risk(n= 16) | *P* |
| Preoperative complications |  |  | 0.071 |
| No | 41 (56.2) | 5 (31.3) |  |
| Yes | 32 (43.8) | 11 (68.8) |  |
| Preoperative T stage |  |  | 0.591 |
| T1 | 28 (38.4) | 7 (43.8) |  |
| T2 | 28 (38.4) | 4 (25.0) |  |
| T3 | 15 (20.5) | 5 (31.2) |  |
| T4a | 2 (2.7) | 0 (0.0) |  |
| Preoperative hemoglobin | 127.14 (11.883) | 122.81 (17.298) | 0.264 |
| Preoperative AST | 22.78 (5.012) | 33.63 (29.344) | 0.063 |
| prei_wavelet_LLL_firstorder_Energy^*^ | -0.204 (0.815) | 0.477 (0.892) | 0.001* |
| prei_wavelet_LHH_gldm_DependenceNonUniformityNormalized^*^ | 0.069 (0.787) | -0.496 (0.451) | 0.002* |
| intra_wavelet_LLH_firstorder_Median | -0.028 (1.507) | -0.188 (0.684) | 0.661 |
| prei_wavelet_LLL_firstorder_TotalEnergy^*^ | -0.188 (0.919) | 0.368 (0.998) | 0.012* |
| prei_original_gldm_DependenceVariance^*^ | -0.162 (0.945) | 0.261 (0.390) | 0.008* |
| prei_wavelet_LHH_glcm_InverseVariance | 0.154 (0.873) | 0.010 (0.529) | 0.417 |
| prei_wavelet_LLH_glszm_GrayLevelNonUniformity^*^ | -0.243 (0.792) | 0.593 (1.009) | 0.001* |
| prei_wavelet_HLL_glcm_Imc1^*^ | -0.176 (0.938) | 0.398 (0.690) | 0.018* |
| prei_wavelet-LLH_glszm_GrayLevelNonUniformityNormalized^*^ | 0.071 (0.859) | -0.422 (0.804) | 0.015* |
| prei_original_glszm_LargeAreaLowGrayLevelEmphasis | 0.025 (1.318) | -0.083 (0.190) | 0.080 |
| prei_wavelet-LLL_glrlm_RunEntropy^*^ | -0.013 (0.818) | 0.599 (0.801) | 0.012* |
| prei_wavelet_LLH_gldm_SmallDependenceLowGrayLevelEmphasis | 0.191 (1.144) | -0.332 (0.548) | 0.069 |
| prei_wavelet_HHH_glrlm_LowGrayLevelRunEmphasis | 0.114 (1.077) | 0.308 (1.105) | 0.281 |
| intra_wavelet_HLH_gldm_SmallDependenceLowGrayLevelEmphasis | -0.023 (0.906) | -0.022 (0.486) | 0.305 |
| prei_wavelet_LLL_gldm_DependenceEntropy^*^ | -0.250 (0.903) | 0.816 (0.618) | <0.001* |
| prei_wavelet_HLH_glszm_SizeZoneNonUniformity^*^ | -0.215 (0.564) | 0.153 (0.608) | 0.007* |
| intra_wavelet_HHL_glszm_SmallAreaEmphasis | -0.260 (1.132) | -0.144 (0.659) | 0.940 |

Continuous variables were represented as mean with standard deviation and categorical variables were summarized by count and percentage. Preoperative complications typically involve chronic obstructive pulmonary disease, coronary heart disease and other diseases; AST, aspartate aminotransferase.

**P* < 0.05**Supplementary Table 6.** Validation of the optimal RSF model based on clinical features alone.

| **Model** | **6 to 54 mo** | | **tAUC at 1-year**  **(95%CI)** | **tAUC at 3-year**  **(95%CI)** |
| --- | --- | --- | --- | --- |
|  | **iBS** | **iAUC** |  |  |
| Clin-RSF | 0.154 | 0.709 | 0.765 (0.700-0.926) | 0.701 (0.605-0.926) |

The optimal RSF model was developed using clinical features selected by multivariate Cox analysis. iBS, integrated brier score; iAUC, integrated area under the curve; tAUC, time-dependent area under the curve; CI, confidence interval; Clin, clinical features; RSF, random survival forest.

**Supplementary Table 7.** Validation of the optimal GBDT model on T1 and T2-4a stage subgroups.

| **T stage** | **6 to 54mo** | | **tAUC at 1-year**  **(95%CI)** | **tAUC at 3-year**  **(95%CI)** |
| --- | --- | --- | --- | --- |
|  | **iBS** | **iAUC** |  |  |
| **T1** | 0.130 | 0.700 | 0.666(0.471-0.712) | 0.675(0.471-0.732) |
| **T2-4a** | 0.155 | 0.908 | 0.757(0.719-0.769) | 0.787(0.719-0.875) |

The optimal GBDT model used clinical features and intra- and peritumoral features via VIMP > 0.01. GBDT, gradient boosting decision tree. iBS, integrated brier score; iAUC, integrated area under the curve; tAUC, time-dependent area under the curve.


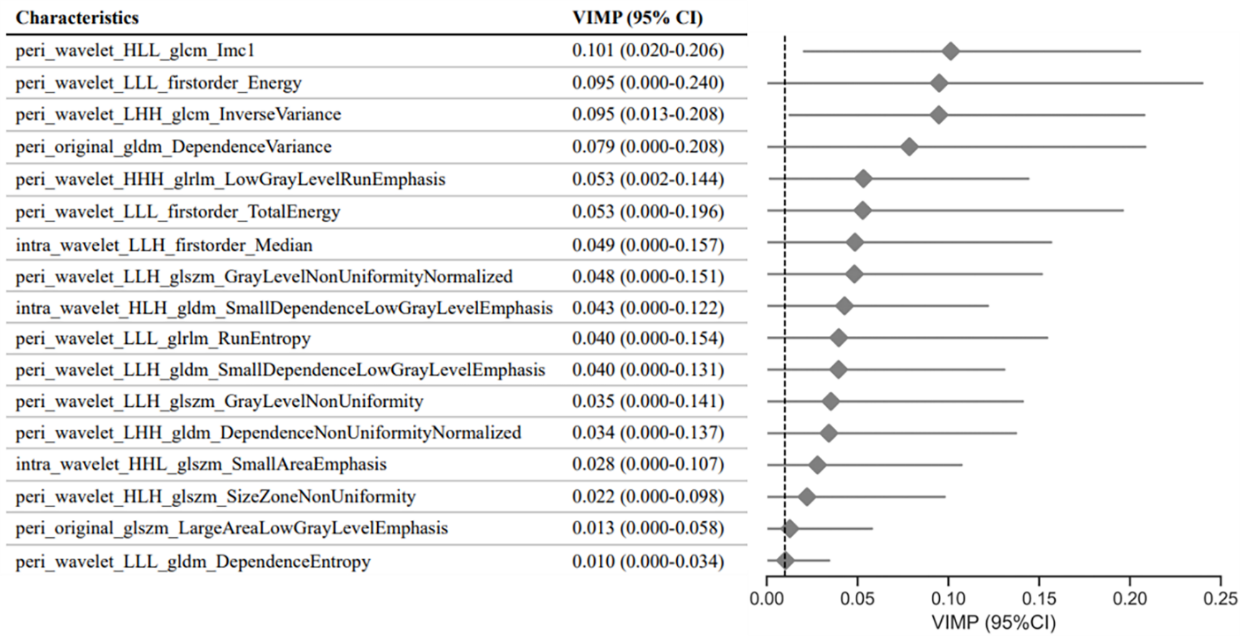


**Supplementary Figure 1.** The intra- and peritumoral radiomic features selected by the method of VIMP > 0.01.

In this Figure, the VIMP was validated by the bootstrapping method with 1000 repetitions and shown as a mean with 95% CI. The features with VIMP greater than 0.01 were included in the final modeling. VIMP, variable importance; CI, confidence interval.


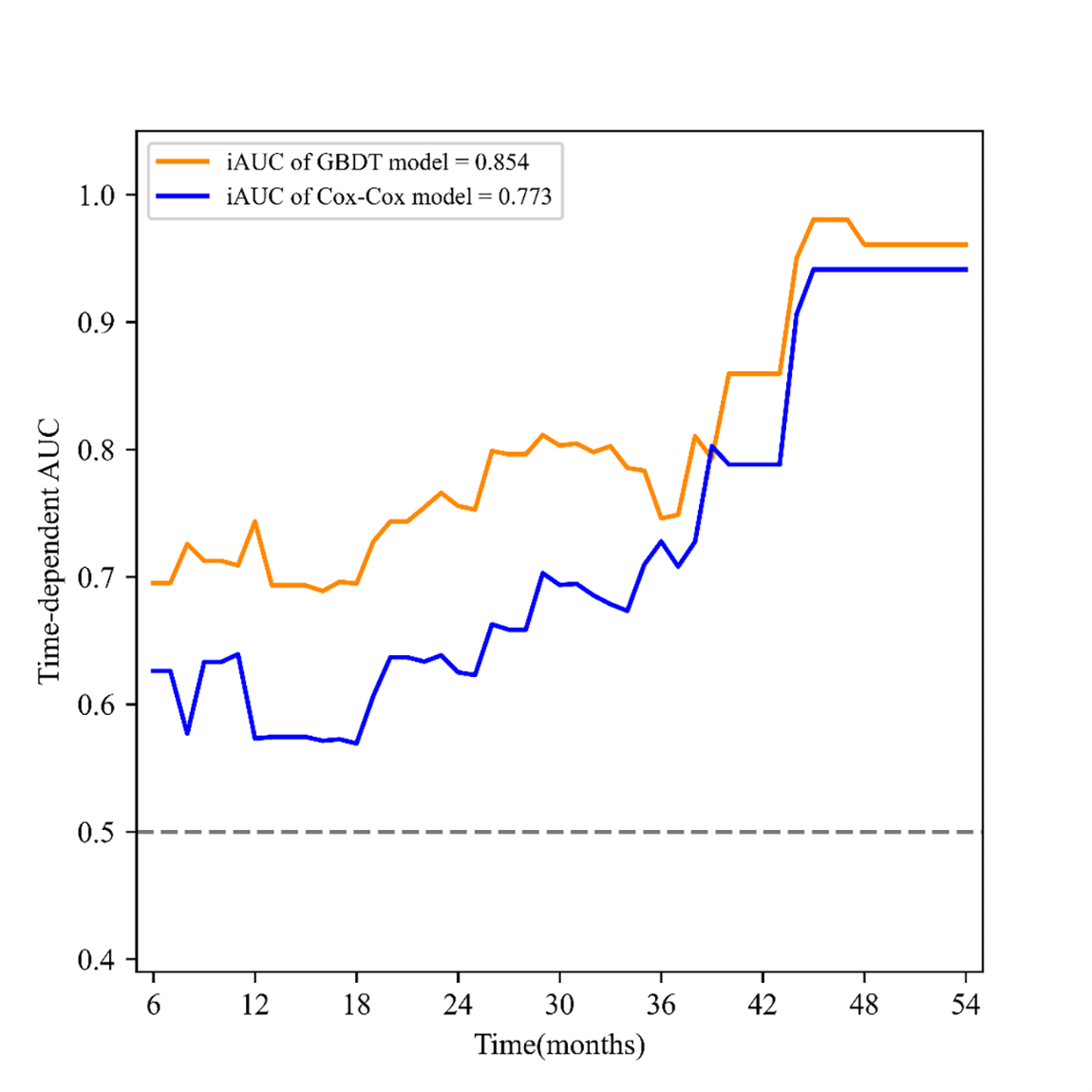


**Supplementary Figure 2.** Time-dependent ROC of the optimal GBDT and Cox regression models in the test set.

The GBDT models used intra- and peritumoral radiomic features via VIMP > 0.01 method with significant clinical characteristics. The Cox-Cox model used intra- and peritumoral radiomic features selected by the Cox regression method with significant clinical characteristics. VIMP, variable importance determined by bootstrap method repeated 1000 times; VIMP > 0.01, features with an average VIMP greater than 0.01; GBDT, gradient boosting decision tree; iAUC, the integrated area under the curve.
